# The longitudinal associations of reading, writing and screen time with myopia at age 9 years among children from the GUSTO birth cohort

**DOI:** 10.1111/aos.70009

**Published:** 2025-10-01

**Authors:** Fan Wu, Chen‐Hsin Sun, Hla Myint Htoon, Jonathan Y. Bernard, Fabian Yap, Yih‐Chung Tham, Charumathi Sabanayagam, Seang‐Mei Saw

**Affiliations:** ^1^ Saw Swee Hock School of Public Health National University of Singapore Singapore Singapore; ^2^ Singapore Eye Research Institute Singapore National Eye Centre Singapore Singapore; ^3^ Department of Ophthalmology Yong Loo Lin School of Medicine, National University of Singapore Singapore Singapore; ^4^ Inserm, INRAE, Centre for Research in Epidemiology and StatisticS (CRESS) Université Paris Cité and Université Sorbonne Paris Nord Paris France; ^5^ Inserm, Desbrest Institute of Epidemiology and Public Health (IDESP) Université de Montpellier Montpellier France; ^6^ Ophthalmology and Visual Sciences Academic Clinical Programme Duke‐NUS Medical School Singapore Singapore; ^7^ Endocrinology Service, Department of Pediatrics KK Women's and Children's Hospital Singapore Singapore; ^8^ Lee Kong Chian School of Medicine Nanyang Technological University Singapore Singapore

**Keywords:** children, myopia, near work, reading and writing, screen time

## Abstract

**Purpose:**

To investigate the associations between paper‐based reading and writing time, screen‐based time at ages 2, 3, 6 and 9 years and myopia at age 9 in the GUSTO birth cohort.

**Methods:**

The GUSTO study recruited pregnant women from two Singapore public maternity hospitals between 2009 and 2010. Parent‐reported reading and writing time, screen time and outdoor time were collected at ages 2, 3, 6 and 9 years. Cycloplegic autorefraction and axial length were measured at age 9. Myopia was defined as a spherical equivalent ≤−0.5 D. Associations between near work exposures and myopia were examined using multivariable regression with generalised estimating equations.

**Results:**

Among 471 children (942 eyes), 37.3% were myopic at age 9 years. Greater reading and writing time at ages 6 and 9 were associated with higher odds of myopia at age 9 (OR [95% CI] = 1.20 [1.02–1.42] and 1.11 [1.02–1.22] per h/day, respectively). Children spending >3 h/day reading and writing at age 9 had 76% higher odds of myopia than those spending ≤3 h/day (OR = 1.76, 95% CI: 1.08–2.85). Reading and writing time at ages 2 and 3 years, and screen time at all age groups showed no significant association with myopia at age 9.

**Conclusions:**

Traditional paper‐based reading and writing, but not screen time, were associated with myopia in Singaporean children. Future studies with larger samples and objective screen time measures are needed to evaluate the distinct role of screen time in myopia.

## 1 INTRODUCTION

The global prevalence of myopia has increased substantially over the past few decades, with projections indicating that by 2050 about half of the world's population will be myopic and approximately 10% will have high myopia (Holden et al., 2016; Pan et al., 2012; World Health Organization, 2016). In East and Southeast Asian countries, the prevalence of myopia among young people has reached alarming levels, with rates as high as 80–90% (Morgan et al., 2012; Wu et al., 2016). Early onset of myopia increases the risk of developing high myopia and ocular complications later in life (Chua et al., 2016).

Prolonged near work and reduced outdoor activities are two key environmental risk factors for myopia (Lingham et al., 2020; Morgan et al., 2021). Near work refers to activities requiring sustained visual focus on close objects, such as traditional paper‐based reading/writing, drawing and using digital screen‐based devices (mobile phones, computers, tablets) (Gajjar & Ostrin, 2022). In Huang et al.'s meta‐analysis of 10,384 children aged 6 to 18 years, greater near work showed a positive association with myopia (odds ratio [OR] = 1.14, 95% confidence interval [CI]: 1.08–1.20) and myopic children read for 0.66 more hours weekly than non‐myopic peers (Huang et al., 2015). A cross‐sectional analysis of data from a consortium of studies from Singapore, China and Hong Kong (*n* = 12,241, ages 4 to 18 years) reported that increased reading and writing time was associated with myopia (OR = 1.17; 95% CI: 1.11–1.24) (Lanca et al., 2022). The proposed mechanism involves visual blur, which disrupts emmetropisation signals in the retina and stimulates axial elongation, as demonstrated in animal models (Wallman & Winawer, 2004).

With the rapid proliferation of digital devices, screen time has become an increasingly prevalent form of near work among children in the past decade (Gajjar & Ostrin, 2022; Morgan et al., 2021). However, evidence regarding its association with myopia remains mixed (Foreman et al., 2021; Lanca & Saw, 2020). Zong et al.'s meta‐analysis (Zong et al., 2024) of 19 studies (*n* = 102 360; ages 0 to 19 years) supported an association between high screen exposure and myopia, with pooled ORs of 2.39 (95% CI: 2.07–2.76) in cohort studies and 2.24 (95% CI: 1.47–3.42) in cross‐sectional studies. Conversely, findings from other studies have reported no significant association (Berticat et al., 2020; Lanca et al., 2022; Lanca & Saw, 2020).

This inconsistency in findings represents a significant research gap, particularly in comparing the effects of different types of near work on myopia. Few studies have compared the impact of different types of near work, paper‐based reading/writing versus screen‐based activities, on myopia within the same population (Enthoven et al., 2020; Harrington et al., 2019; Lanca et al., 2022). As near work patterns are changing from paper‐based to screen‐based activities, the effects of limited paper‐based reading and writing time on myopia should also be evaluated while accounting for potential confounding introduced by time spent outdoors (Xiong et al., 2017).

In this study, we aimed to investigate the associations between different types of near work at ages 2, 3, 6 and 9 years, with myopia outcomes at age 9 among children from the Growing Up in Singapore Towards healthy Outcomes (GUSTO) cohort. Age 9 was selected as the outcome time point based on findings from the Singapore Cohort of the Risk factors for Myopia (SCORM) cohort, which reported a mean myopia onset age of 8.0 years, supporting its relevance for evaluating early myopia development (Chua et al., 2016).

## 2 METHODS

### 2.1 Study population

**FIGURE 1:**
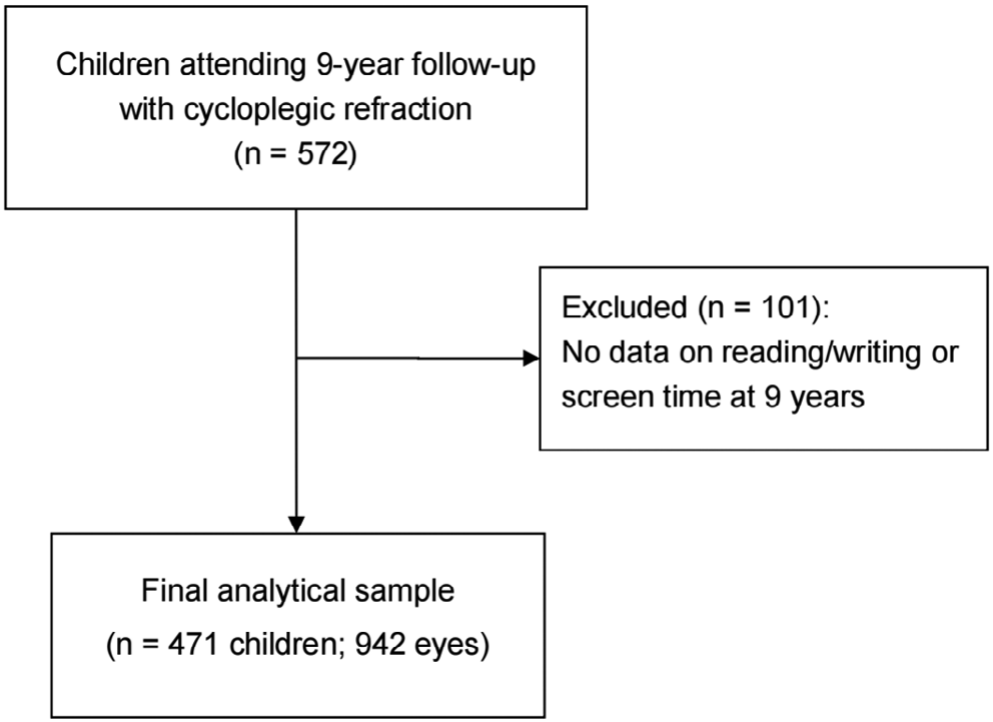
The flowchart of participant inclusion at the 9‐year ocular assessment.

The GUSTO study recruited pregnant women aged ≥18 years from two major public maternity hospitals in Singapore from 2009 to 2010 (Soh et al., 2013). This longitudinal study evaluated children's paper‐based reading/writing time and screen time at ages 2, 3, 6 and 9 years, with an ocular assessment at a follow‐up visit at age 9. A total of 572 participants had complete data on cycloplegic refraction. Of these, 471 children with both cycloplegic refraction data and at least one measurement of reading/writing time or screen time at age 9 were included in the final analytical cohort. We excluded 101 children who provided no information about reading/writing or screen exposure at the 9‐year assessment. The flow of participants from enrolment to the final analytical cohort is shown in Figure 1. Participating families provided written informed consent and child assent. The research protocol was approved by the National Healthcare Group Domain Specific Review Board and the SingHealth Centralised Institutional Review Board. All procedures conformed to the Declaration of Helsinki principles.

### 2.2 Assessment of reading/writing time and screen time

The GUSTO Child Eye Questionnaire, completed by parents, was administered during follow‐up assessments of children ages 2, 3, 6 and 9. The questionnaire collected data on screen time and reading/writing time. Parents answered the following question: ‘How much time per day on average would you estimate your child doing the following activities in the past 1 month?’ For each activity, parents reported time spent in hours and minutes (in 5‐minute increments), separately for weekdays (Monday to Friday) and weekends (Saturday to Sunday). The reading/writing time was assessed through the item ‘Self‐reading and/or writing or shown a book by caregiver’. At ages 2 and 3, parent‐reported reading/writing time primarily reflected shared reading with adults, as children at these ages rarely engage in sustained independent reading. The questionnaire did not differentiate between shared and independent reading. Screen time was measured through two items: ‘Uses a computer/plays on computers’ and ‘Plays handheld video games or uses handheld devices like handphone to play games/watch videos (e.g. gameboy, handphone games)’. Television time was assessed through a separate question but was not included as part of screen time, as television viewing is generally not considered near work.

### 2.3 Assessment of covariates

Parents provided information about their child's sex, ethnicity (categorised as Chinese, Malay and Indian), parental myopia status (none or at least one myopic parent) and maternal education levels (high school or below, diplomas/certificates, university or above). Time spent outdoors was assessed by parents' reports of their child's daily outdoor activities (Xiong et al., 2017). For each activity, the weighted average daily exposure in hours per day (h/day) was calculated using the formula: (5 × h/day on weekdays + 2 × h/day on weekends) / 7. This calculation provided a composite measure reflecting typical daily exposure throughout the week.

### 2.4 Ocular examination and definition of outcomes

Ocular examinations were conducted during the 9‐year follow‐up visit. Cycloplegic refraction was measured using an autorefractor (Canon RK‐5/RK‐F2, Canon, Tokyo, Japan). Cycloplegia was administered by instillation of 1% cyclopentolate hydrochloride, three drops at 5‐minute intervals. Autorefraction measurements were taken at least 30 minutes after initial instillation and only when the pupil had dilated to a minimum of 6 mm. Spherical equivalent (SE) was obtained through the formula: sphere (D) + (cylinder (D)/2). Axial length (AL) was assessed by an optical biometer (IOL Master 500, Carl Zeiss‐Meditec, Oberkochen, Germany). The primary outcome measures at the 9‐year examination were myopia status (defined as SE ≤–0.5 D), SE and AL.

### 2.5 Statistical analysis

We analysed data from 471 children (942 eyes). Right eye data were used for descriptive analyses and figures to maintain comparability with previous studies. For association analyses, both eyes were included. To improve statistical efficiency and account for the intra‐subject correlation of paired eyes, we fitted multivariable regression models using Generalised Estimating Equations (GEE) with an exchangeable working correlation and robust (sandwich) standard errors, clustering on child (Fan et al., 2011). Logistic regression with GEE was employed for the binary myopia outcome, and linear regression with GEE was used for the continuous SE and AL outcomes. Each ocular outcome (myopia, SE, AL) was analysed in a separate model. SE and AL were not included as covariates in each other's models to avoid collinearity. Model building followed a manual backward selection approach of potential covariates. Covariates were retained if they were statistically significant (*p* < 0.05) or established as confounders in childhood myopia research based on prior literature. The following potential confounders were examined: sex, ethnicity, maternal education, parental myopia status and children's time spent outdoors, selected based on prior literature and biological plausibility. Final multivariable regression models were adjusted for sex, ethnicity, parental myopia status and time spent outdoors (Czepita et al., 2007; Jiang et al., 2020; Pan et al., 2013; Ramamurthy et al., 2015). Maternal education was not adjusted for as its inclusion did not materially improve model fit (based on adjusted *R* ^2^ and log‐likelihood). All statistical analyses were performed using two‐sided tests, with statistical significance defined as *p* < 0.05, using Stata v15 (StataCorp, College Station, TX, USA).

## 3 RESULTS

The analysis included 471 children (942 eyes). Of these, 52.4% were boys, 55.8% were of Chinese ethnicity, 29.3% Malay and 14.9% Indian. Most children (76.2%) had at least one myopic parent. At age 9 years, 351 eyes (37.3%) were myopic (SE ≤−0.5 D), with a mean SE of −0.3 ± 1.7 D and a mean AL of 23.4 ± 1.0 mm. In total, 40.6% of children had at least one myopic eye. The agreement in myopia status between both eyes was 93.0%; 34.0% of children had bilateral myopia, and 6.6% had unilateral myopia.

Reading/writing time and screen time increased with age (Table 1). Reading and writing time (mean ± standard deviation [SD]) increased from 0.6 ± 0.9 h/day at age 2 years to 2.2 ± 2.1 h/day at age 9 years. Screen time (mean ± SD) increased from 0.8 ± 1.0 h/day at age 2 years to 2.0 ± 1.8 h/day at age 9 years.

**TABLE 1:** Reading/writing and screen time across different age groups in children from the GUSTO cohort (*n* = 471).

Age group (years)	Reading/writing time (h/day)^a^		Screen time (h/day)^a^	
	*n*	Mean SD	*n*	Mean SD
2	418	0.6 ± 0.9	448	0.8 ± 1.0
3	424	0.6 ± 0.8	447	1.1 ± 1.3
6	425	1.3 ± 1.2	431	1.3 ± 1.3
9	471	2.2 ± 2.1	456	2.0 ± 1.8

*Note*: Analyses are based on the right eye.

Abbreviation: SD, standard deviation.

^a^ Daily averages (h/day) were computed using the formula: [(5× h/day on weekdays +2× h/day on weekends)/7].

**FIGURE 2:**
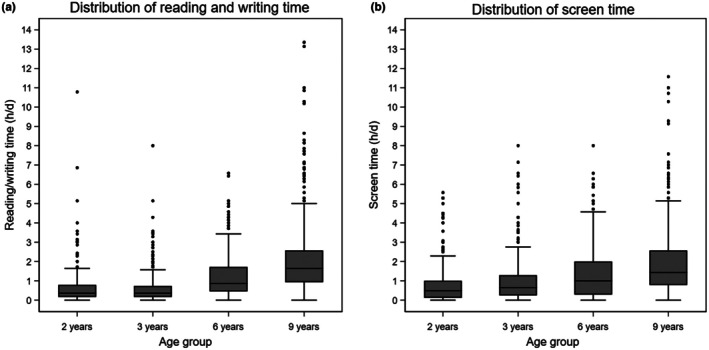
Distribution of daily reading/writing time and screen time across different age groups. Box plots showing (a) reading/writing time and (b) screen time in hours per day for children aged 2, 3, 6 and 9 years. Both activities increase with age, with pronounced increases observed in the 6‐ and 9‐year age groups. Analyses are based on the right eye.

Figure 2 shows the distribution of reading/writing time and screen time across different age groups. Both distributions were right‐skewed, and most children spent less than 3 h/day on these activities for all ages. The proportion of children spending over 3 h/day on reading and writing increased from 1.9% at ages 2 and 3 to 9.4% at age 6 and 18.5% at age 9. Similarly, the proportion of children using screens for more than 3 h/day increased from 3.8% at age 2 to 18.2% at age 9.

Before examining the effects of reading and screen time, we assessed the covariates included in the multivariable models (Table 2). Male sex was associated with longer AL but not with myopia or SE. Compared with Chinese children, Malay children had lower odds of myopia, less myopic SE and shorter AL. Indian children showed a similar pattern, but only the association with SE reached significance. Having at least one myopic parent was associated with higher odds of myopia, with a borderline association with more myopic SE. Greater outdoor time was protective against myopia and associated with less myopic SE, with no significant association with AL.

**TABLE 2:** Associations of covariates with myopia, SE and AL at age 9 years from multivariable GEE models in the GUSTO cohort (eyes = 942; *n* = 471).

Covariate	Myopia (SE ≤−0.5 D)		SE (D)		AL (mm)	
	OR [95% CI]	*p* ^a^	*β* [95% CI]	*p* ^b^	*β* [95% CI]	*p* ^b^
Sex						
Female	Reference	–	Reference	–	Reference	–
Male	0.98 [0.67, 1.45]	0.93	−0.11 [−0.42, 0.21]	0.51	0.67 [0.47, 0.87]	<0.001
Ethnicity						
Chinese	Reference	–	Reference	–	Reference	–
Malay	0.47 [0.29, 0.75]	0.002	0.54 [0.19, 0.88]	0.002	−0.34 [−0.56, −0.11]	0.004
Indian	0.58 [0.31, 1.08]	0.09	0.49 [0.07, 0.91]	0.022	−0.19 [−0.49, 0.12]	0.23
Parental myopia						
None	Reference	–	Reference	–	Reference	–
At least one parent	1.75 [1.07, 2.86]	0.026	−0.35 [−0.71, 0.01]	0.057	0.13 [−0.11, 0.38]	0.29
Outdoor time (h/day)	0.86 [0.75, 0.99]	0.033	0.14 [0.04, 0.25]	0.005	−0.05 [−0.12, 0.03]	0.22

Abbreviations: AL, axial length; CI, confidence interval; OR, odds ratio; SE, spherical equivalent.

^a^ Generalised estimating equations (GEE)‐based logistic regression accounting for paired‐eye data.

^b^ GEE‐based linear regression accounting for paired‐eye data.

At ages 6 and 9 years, each additional hour of daily reading and writing was associated with higher odds of myopia (OR = 1.20, 95% CI: 1.02–1.42, *p* = 0.028; OR = 1.11, 95% CI: 1.02–1.22, *p* = 0.019, respectively), after adjusting for sex, ethnicity, parental myopia status and time spent outdoors (Table 3). At age 9, a per‐hour increase in reading and writing time showed a significant association with AL (*β* = 0.05 mm, 95% CI: 0.01–0.09, *p* = 0.019). Children whose daily reading and writing time exceeded 3 h/day had 76% higher odds of developing myopia compared to those whose exposure was less than 3 h/day (OR = 1.76, 95% CI: 1.08–2.85, *p* = 0.023). Reading and writing time at ages 2 and 3 years was not associated with myopia, SE or AL at 9 years.

**TABLE 3:** Associations between daily reading and writing time and myopia, SE and AL from age 2 to 9 years among children from the GUSTO cohort (eyes = 942; *n* = 471).

Age group (years)		Myopia (SE ≤−0.5 D)		SE (D)		AL (mm)	
	Reading and writing time (h/day)	OR [95% CI]	*p* ^a^	*β* [95% CI]	*p* ^b^	*β* [95% CI]	*p* ^b^
2	Continuous^c^	1.12 [0.91, 1.37]	0.27	−0.06 [−0.22, 0.11]	0.50	0.04 [−0.03, 0.12]	0.28
	0–3 h	Reference	–	Reference	–	Reference	–
	>3 h	0.94 [0.19, 4.61]	0.94	0.07 [−0.79, 0.94]	0.87	0.31 [−0.14, 0.75]	0.18
3	Continuous^c^	0.78 [0.59, 1.03]	0.09	0.09 [−0.06, 0.23]	0.26	−0.06 [−0.16, 0.04]	0.22
	0–3 h	Reference	–	Reference	–	Reference	–
	>3 h	0.27 [0.03, 2.10]	0.21	0.50 [−0.07, 1.07]	0.09	−0.05 [−0.40, 0.31]	0.78
6	Continuous^c^	1.20 [1.02, 1.42]	0.028	−0.15 [−0.32, 0.01]	0.06	0.09 [−0.01, 0.18]	0.07
	0–3 h	Reference	–	Reference	–	Reference	–
	>3 h	1.66 [0.86, 3.19]	0.13	−0.46 [−1.12, 0.20]	0.18	0.16 [−0.21, 0.52]	0.40
9	Continuous^c^	1.11 [1.02, 1.22]	0.019	−0.07 [−0.13, −0.0002]	0.05	0.05 [0.01, 0.09]	0.019
	0–3 h	Reference	–	Reference	–	Reference	–
	>3 h	1.76 [1.08, 2.85]	0.023	−0.41 [−0.86, 0.05]	0.08	0.24 [−0.03, 0.51]	0.08

*Note*: Multivariable models adjusted for sex, ethnicity, parental myopia status and time spent outdoors.

Abbreviations: AL, axial length; CI, confidence interval; OR, odds ratio; SE, spherical equivalent.

^a^ GEE‐based logistic regression accounting for paired‐eye data.

^b^ GEE‐based linear regression accounting for paired‐eye data.

^c^ Continuous exposure analysed as hours per day (h/day).

**FIGURE 3:**
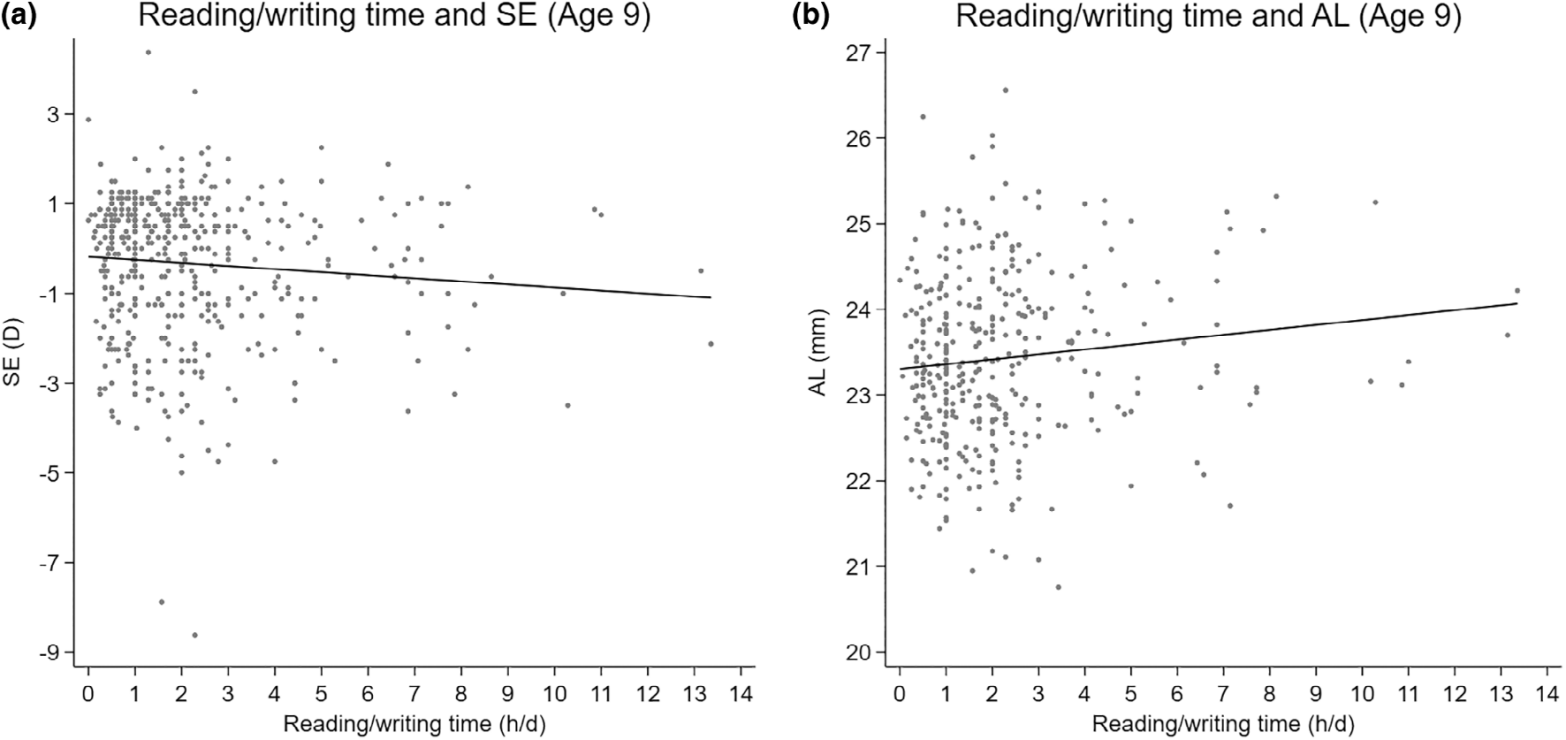
Relationship between daily reading/writing time and ocular parameters among 9‐year‐old children (*n* = 471). Scatter plots show the association between daily reading/writing time (h/day) and (a) spherical equivalent (SE) and (b) axial length (AL) among 471 children based on the right eye. Linear regression lines are shown.

Figure 3 illustrates the relationships between daily reading and writing time at 9 years and ocular parameters based on right eye data (*n* = 471). Figure 2a reveals a trend towards more myopic SE with increasing reading and writing time, although this relationship does not reach statistical significance (*β* = −0.07, 95% CI: −0.14 to 0.01, *p* = 0.08). Figure 2b shows a significant positive association between reading and writing time and AL, with *β* = 0.06 (95% CI: 0.01 to 0.11, *p* = 0.020).

In contrast to reading and writing time, after adjusting for sex, ethnicity, parental myopia status and time spent outdoors, screen time measured at all ages was not associated with myopia, SE or AL (Table 4).

**TABLE 4:** Associations between daily screen time and myopia, SE and AL from age 2 to 9 years among children from the GUSTO cohort (eyes = 942; *n* = 471).

Age group (years)		Myopia (SE ≤−0.5 D)		SE (D)		AL (mm)	
	Screen time (h/day)	OR [95% CI]	*p* ^a^	*β* [95% CI]	*p* ^b^	*β* [95% CI]	*p* ^b^
2	Continuous^c^	0.89 [0.73, 1.09]	0.25	0.13 [−0.01, 0.26]	0.06	−0.04 [−0.13, 0.05]	0.42
	0–3 h	Reference	–	Reference	–	Reference	–
	>3 h	0.61 [0.18, 2.03]	0.42	0.45 [−0.13, 1.03]	0.13	−0.30 [−0.75, 0.15]	0.19
3	Continuous^c^	1.14 [0.97, 1.34]	0.11	−0.10 [−0.24, 0.03]	0.14	0.07 [−0.01, 0.14]	0.09
	0–3 h	Reference	–	Reference	–	Reference	–
	>3 h	1.74 [0.78, 3.90]	0.18	−0.12 [−0.76, 0.53]	0.73	−0.03 [−0.43, 0.36]	0.87
6	Continuous^c^	1.08 [0.91, 1.28]	0.37	−0.05 [−0.21, 0.11]	0.52	0.05 [−0.05, 0.15]	0.32
	0–3 h	Reference	–	Reference	–	Reference	–
	>3 h	1.29 [0.63, 2.65]	0.49	−0.20 [−0.86, 0.46]	0.55	0.10 [−0.31, 0.51]	0.64
9	Continuous^c^	1.01 [0.90, 1.12]	0.92	0.05 [−0.04, 0.13]	0.26	−0.01 [−0.06, 0.04]	0.71
	0–3 h	Reference	–	Reference	–	Reference	–
	>3 h	1.43 [0.85, 2.40]	0.18	−0.04 [−0.43, 0.35]	0.84	0.01 [−0.23, 0.26]	0.91

*Note*: Multivariable models adjusted for sex, ethnicity, parental myopia status and time spent outdoors.

Abbreviations: AL, axial length; CI, confidence interval; OR, odds ratio; SE, spherical equivalent.

^a^ GEE‐based logistic regression accounting for paired‐eye data.

^b^ GEE‐based linear regression accounting for paired‐eye data.

^c^ Continuous exposure analysed as hours per day (h/day).

Correlation analysis between reading/writing time and screen time showed a weak but significant positive correlation at ages 2 and 9 years (*r* = 0.17, *p* < 0.001 and *r* = 0.26, *p* < 0.001, respectively), while no significant correlations were observed at ages 3 and 6 years (*r* = 0.02, *p* = 0.65 and *r* = 0.07, *p* = 0.16, respectively). The stronger correlation at age 9 suggests that once children start formal education, those who spend more time reading and writing also tend to spend more time on screen‐based activities. We also examined the association between total near work (combined reading/writing and screen time) and myopia in each age group. At age 6, total near work showed a significant association with myopia (OR = 1.12, 95% CI: 1.01–1.24, *p* = 0.034) and AL (*β* = 0.06 mm, 95% CI: 0.01–0.12, *p* = 0.025). No significant associations were observed at ages 2, 3 and 9 (all *p* > 0.05). These findings suggest that age 6 may represent a particularly sensitive period for near work effects, coinciding with the start of formal education.

## 4 DISCUSSION

### 4.1 Main findings

The GUSTO birth cohort uses a longitudinal design with repeated measurements of near work exposure from 2 to 9 years. These longitudinal data enable us to examine temporal exposure patterns and identify critical periods when different types of near work may impact myopia development. In multivariable models, traditional paper‐based reading and writing time at ages 6 and 9 were associated with myopia and additionally with increased AL at age 9. By contrast, screen time at all age groups showed no significant association with myopia, SE or AL.

### 4.2 Paper‐based near work

The significant association between reading and writing time at ages 6 and 9 years and myopia reaffirms the role of traditional near work activities in myopia development (Huang et al., 2015; Lanca et al., 2022; Wallman & Winawer, 2004), consistent with a meta‐analysis of 254 037 participants aged 6–39 years, which reported higher odds of myopia with near work (OR = 1.26, 95% CI: 1.18–1.34), particularly among children (OR = 1.31, 95% CI: 1.21–1.42) (Dutheil et al., 2023). Children with longer reading/writing time at age 9 had greater AL. Axial elongation is a well‐recognised structural characteristic of myopia. However, in children, AL alone does not fully determine refractive status, as corneal and lenticular power also change during emmetropisation (Ip et al., 2007; Mutti et al., 2005). Accordingly, we analysed AL in parallel with myopia status and SE rather than as its sole determinant. In categorical analyses, children who spent >3 h/day on reading and writing at age 9 had 76% higher odds of myopia than peers with ≤3 h/day. Similar patterns were observed in the UK ALSPAC cohort (*n* = 2005), where longer reading (1–2 h/day or >3 h/day) at age 8 to 9 years was associated with a 32% increased odds of myopia at age 11 to 15 years (OR = 1.32, 95% CI: 1.02–1.71) (Guggenheim et al., 2012). The mechanism may involve hyperopic defocus combined with accommodation lag during prolonged near work, stimulating axial elongation, which is exacerbated by the lack of intermittent breaks (Ip et al., 2008; Wallman & Winawer, 2004). In a 3‐year follow‐up of Finnish schoolchildren (*n* = 238), greater near work and shorter reading distance were associated with faster myopic progression, whereas higher accommodative stimulus was not (Pärssinen & Lyyra, 1993). In historical cross‐sectional analyses of 4961 Finnish children, more near work increased myopia risk, which was partly offset by additional outdoor time, although the protective effect attenuated at the highest levels of near work (Pärssinen & Kauppinen, 2022). In our cohort, most Singaporean children start formal education at age 6, when reading and writing time increased substantially to 1.2 h/day and further to 2.2 h/day at age 9; the association with myopia coincides with this period of intensive educational demands. By contrast, reading and writing time at ages 2 and 3 was minimal (mean 0.6 h/day) and showed no significant association with myopia.

### 4.3 Screen time

In our study, there was no significant association between screen time and myopia (all *p* > 0.05), consistent with studies from Singapore, Germany and Norway and earlier meta‐analyses reporting null associations (Hagen et al., 2018; Lanca et al., 2022; Lanca & Saw, 2020; Schuster et al., 2020; Toh et al., 2019). By contrast, some studies in India, China, Ireland and Denmark have reported positive associations (Guan et al., 2019; Hansen et al., 2020; Harrington & O'Dwyer, 2023; Saxena et al., 2015). Irish and Chinese studies in schoolchildren found that >3 h/day of screen time was significantly associated with myopia (OR [95% CI] = 3.7 [2.1–6.3] and 2.11 [1.20–3.70], respectively) (Harrington et al., 2019; Xie et al., 2020). These inconsistent results may reflect limited statistical power and methodological differences in defining and measuring screen time.

Among the few studies comparing both reading/writing and screen time in the same population, the Generation R study and Sunflower Myopia AEEC study reported associations for reading/writing but not screens, whereas the Irish study found associations for both types of near work (Enthoven et al., 2020; Harrington et al., 2019; Lanca et al., 2022). One possible explanation for the smaller effect of screen time compared with paper‐based reading/writing on myopia is differences in visual demands and usage patterns. Reading/writing requires higher visual acuity for text processing and tends to be continuous, whereas screen use often involves images and dynamic content and is more likely to be interrupted by notifications or application switching, potentially providing intermittent visual rest. The lack of association in our study may reflect a smaller effect size. Studies reporting significant screen effects typically had larger samples, including 1626 children in Ireland, 9616 in India and 19 934 in China (Guan et al., 2019; Harrington & O'Dwyer, 2023; Saxena et al., 2017). The All Babies in Southeast Sweden (ABIS) cohort (*n* = 5200) reported no association between childhood screen time and myopia in young adulthood but found a protective effect of outdoor time at age 8 years, while reading was not significant (Bro & Ludvigsson, 2025). By contrast, our study found significant associations of reading/writing at ages 6 and 9 years with myopia, which may reflect differences between East Asian and Scandinavian populations, educational systems and academic demands during critical developmental periods.

Compared with traditional reading and writing, screen time represents a newer form of near work, particularly on portable devices. As screen‐based near work has partly replaced paper‐based reading and writing, particularly in educational settings, it is important to evaluate screen exposure and myopia. Although direct data in young children are limited, population trends suggest a shift in near work patterns: among U.S. 16‐ to 17‐year‐olds, the proportion reading books or magazines declined from 60% in the late 1970s to 16% in 2016 (Twenge et al., 2019), a trend likely extending to younger groups. Future research should investigate how these changing patterns of near work affect myopia.

### 4.4 Clinical and public health implications

Our findings highlight a sensitive window of visual development during early formal education. During this period, increasing academic demands and intensive near‐visual tasks may disrupt normal refractive development. Vision screening and parental guidance at this stage may help identify and mitigate early myopic changes. Prevention should consider limiting both screen time and the duration of traditional reading and writing; school schedules should also balance near work with outdoor activity. For parents, recognising this critical developmental window may enable timely actions, such as limiting the recreational use of screen‐based devices.

### 4.5 Limitations

This prospective birth cohort with repeated measures of near work at ages 2, 3, 6 and 9 years enabled the assessment of associations between age‐specific exposure and myopia prevalence at age 9 years based on cycloplegic refraction. This study has several limitations. First, reading/writing and screen time were parent‐reported, which may introduce exposure misclassification bias. Second, our sample size may have been insufficient to detect small effects, particularly for screen time. Third, at ages 2–3 years, reading exposure was mainly based on shared reading with adults, which likely imposes lower accommodative demands than independent reading. As our questionnaire did not differentiate between shared and independent reading, this may have contributed to misclassification and weaker associations at younger ages.

### 4.6 Future research

Larger longitudinal and interventional studies testing digital viewing habits are essential to clarify the role of screen time in myopia development. Objective measures of near work should be employed to address the limitations of self‐reported measures, including wearable sensors for viewing distance and duration, eye tracking and device usage analytics to quantify both traditional and screen‐based activities. In addition, research examining qualitative aspects of screen use not captured in our analysis is also necessary, including different types of screen content (educational versus entertainment), intermittent versus continuous usage patterns, viewing angles and postural differences between device types (downward gaze with smartphones and tablets versus straight‐ahead viewing with monitors and televisions), and the effects of screen brightness.

## 5 CONCLUSION

Our results demonstrate that traditional paper‐based reading and writing were associated with myopia outcomes in young children in the GUSTO cohort. Larger longitudinal and interventional studies using objective measures of screen exposure are needed to develop effective prevention strategies.
